# Effects of Historical Isolation and Current River Fragmentation on the Genetic Structure of a Freshwater Fish

**DOI:** 10.1002/ece3.74633

**Published:** 2026-11-09

**Authors:** Jon Garrastatxu, Maite Arroita, Simon Blanchet, Arturo Elosegi, Jérôme G. Prunier, Aitor Larrañaga

**Affiliations:** ^1^ Department of Plant Biology and Ecology, Faculty of Science and Technology University of the Basque Country (EHU) Leioa Spain; ^2^ CNRS, Station d'Écologie Théorique et Expérimentale (UAR 2029–SETE) Moulis France; ^3^ ADENEKO Saint‐Girons France

**Keywords:** connectivity, genetic structure, microsatellite, *Phoxinus bigerri*, population isolation

## Abstract

Habitat fragmentation is a major threat to freshwater biodiversity. In river networks, the dendritic structure of drainage systems can compound the effects of barriers to dispersal, which have greatly increased as a result of anthropogenic activities. Yet, disentangling the relative contributions of these natural and anthropogenic barriers remains challenging. We assessed how historical catchment isolation and contemporary river fragmentation jointly shape the genetic structure of a low‐dispersal freshwater fish, the Pyrenean minnow (*Phoxinus bigerri*), across northern Spain by genotyping 280 individuals from five independent catchments at 26 microsatellite loci and analysing genetic structure at regional and within‐catchment scales using STRUCTURE. The effects of fragmentation metrics on pairwise genetic differentiation were quantified with Maximum Likelihood Population Effects mixed models, and demographic histories were reconstructed using BOTTLENECK and DIYABC‐RF. Strong genetic differentiation among catchments was consistent with long‐term isolation, while pronounced substructure within several catchments suggested restricted contemporary connectivity. Among fragmentation metrics, the number of barriers between river reaches emerged as the strongest predictor of genetic differentiation, whereas cumulative barrier height and river distance showed weaker effects. Demographic analyses revealed signatures of recent bottlenecks, likely dating to the post‐medieval period and coinciding with intensified human modification of river networks. Together, these findings suggest that contemporary river fragmentation may reinforce genetic differentiation superimposed on older patterns of catchment isolation, highlight the pervasive impact of barriers on connectivity and provide insights relevant for the conservation and management of fragmented freshwater ecosystems.

## Graphical abstract

We investigated how historical isolation and contemporary fragmentation shape genetic structure in *Phoxinus bigerri* across northern Spain. Microsatellite data reveal strong among‐catchment differentiation driven by historical processes, alongside recent bottlenecks and within‐catchment structuring associated with barrier density. These results demonstrate how recent human impacts amplify long‐term patterns of genetic isolation in riverine systems.

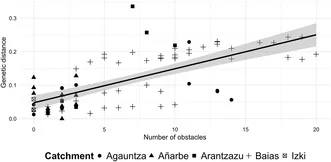

## 1 Introduction

Habitat isolation has important ecological and evolutionary consequences, and it is further exacerbated by habitat fragmentation driven by diverse natural and anthropogenic processes (Cheptou et al. 2017). Rivers are particularly prone to such effects because they are highly structured ecosystems, with drainage basins functioning as natural units isolated from one another, at least for organisms tightly linked to water, as fish. Over geological timescales, glacial cycles and fluctuations in sea level alternately connected and separated entire basins, leaving strong imprints on the spatial distribution of genetic diversity across freshwater species (April et al. 2013; Davis et al. 2018; Wilson and Bernatchez 1998). At finer scales, the dendritic architecture of river networks creates asymmetric patterns of connectivity, with upstream headwaters being naturally more isolated than downstream reaches. These headwaters, traditionally considered as species‐poor, are now recognised as important sources of diversity at the river‐network scale (Finn et al. 2011). Within catchments, additional natural features such as waterfalls, steep reaches or temporary sections have further shaped genetic differentiation among populations in predictable ways (Paz‐Vinas and Blanchet 2015; Reier et al. 2025). Superimposed on these long‐standing natural barriers are more recent anthropogenic barriers such as dams, weirs and culverts. In Europe alone, more than one million barriers fragment river ecosystems, often isolating populations across relatively short distances and representing a major obstacle to achieving good ecological status in water bodies (Belletti et al. 2020). Fragmentation is expected to intensify by increased hydrological variability linked to climate change (Blanchet et al. 2020; Fuller et al. 2015), as well as by new infrastructures that are projected to be built (Zarfl et al. 2015).

Habitat isolation reduces gene flow and erodes intraspecific genetic diversity, which underpins both ecological performance and evolutionary potential, and is a good predictor of population persistence and conservation status (Hoban et al. 2020; Mastretta‐Yanes et al. 2024; Willoughby et al. 2015). When population sizes and gene flow are critically reduced, especially following demographic bottlenecks, drift dominates allele frequency dynamics, effective population size (Ne) becomes small and genetic erosion accelerates (Chhina et al. 2024). This can lead to the chance loss of functionally important alleles involved in stress resistance or local adaptation (Bijlsma and Loeschcke 2012). Fragmentation can also increase evolutionary divergence because selective pressures may vary in environmentally heterogeneous patches—a process sometimes referred to as evolutionary stochasticity (Fraser et al. 2014). Together, the erosion of genetic variation and the amplification of divergent selective pressures compromise both adaptive fitness and demographic stability, thereby increasing extinction risk (Hedrick and Fredrickson 2010; Kardos et al. 2021). However, the magnitude and detectability of these genetic effects depend on several factors, including population size, species‐specific dispersal capacity, barrier permeability and the time elapsed since isolation. Gene flow may mitigate genetic erosion by reintroducing allelic variation and counteracting genetic drift, provided that sufficient functional connectivity is maintained among populations (Frankham 2015; Sexton et al. 2024). Fragmentation has consequently been associated with increased genetic differentiation in fish (Blanchet et al. 2020), reduced resistance to environmental stress (Baguette et al. 2013) and increased risk of population decline or extinction (Nilsson et al. 2005; Pringle 2003). At broades spatial scales, patterns of genetic differentiation have been shown to reflect postglacial colonisation history, whereas present‐day drainage connectivity and natural barriers such as falls and cascades further influenced differentiation at a smaller spatial scale (Costello et al. 2003). Similarly, the genetic structure of the Italian brook chub (*Squalius lucumonis*) was associated with historical hydrogeological isolation among drainage basins, while differentiation at the within‐basin scale was attributed to more recent barriers and the intermittent hydrology of Mediterranean streams (Rossi et al. 2021). At finer temporal and spatial scales, dams and hydrological alteration have been associated with strong genetic discontinuities in species such as the Roanoke logperch ( *Percina rex* ; Roberts et al. 2013). More recently, a comparative study of low‐head dams showed that contemporary genetic connectivity across barriers depends jointly on obstacle characteristics, local hydrological conditions and fish identity. In *Phoxinus* and *Squalius*, taller obstacles reduced genetic connectivity, whereas high‐magnitude, long‐duration and frequent high‐flow events mitigated this effect; the presence of potential passage structures also improved connectivity across the three fish genera examined (Jamonneau et al. 2026). Thus, genetic responses to anthropogenic barriers can be weak or heterogeneous depending on species traits, demographic history, barrier height and permeability, the local hydrological regime, and the time elapsed since barrier construction (Coleman et al. 2018; Gehri et al. 2021; Jamonneau et al. 2026). Together, these studies indicate that broad‐scale genetic patterns mostly retain the legacy of historical processes, whereas contemporary barriers can further modify connectivity and population structure at finer spatial scales, that is, within catchments. They also demonstrate that regional genetic differentiation cannot, by itself, be attributed exclusively to historical isolation, and that the relative contributions of processes acting over different timescales must be interpreted cautiously.

Life‐history traits are also expected to strongly influence the genetic response of individual species to isolation and fragmentation. Species with limited dispersal capacity, strong reliance on upstream‐downstream movements for spawning, or restricted habitat requirements are especially sensitive to isolation (Coleman et al. 2018). One such species is the Pyrenean minnow (*Phoxinus bigerri*), a small cyprinid (6–8 cm total length) that inhabits the mid‐ and upper reaches of rivers in the northern Iberian Peninsula and southwestern France (Leunda et al. 2010; Palandačić et al. 2020). Because of its limited dispersal capacities, even small barriers could cause severe restrictions on its movements and gene flow. Additional pressures such as habitat degradation and pollution further exacerbate fragmentation effects and threaten its genetic viability (Leunda et al. 2010; Oscoz et al. 1999). Although currently classified as Least Concern by the IUCN (Ford 2024), its demographic and ecological characteristics make the Pyrenean minnow particularly susceptible to inbreeding and drift in fragmented systems. Furthermore, the species is rarely translocated by anglers, which minimises artificial gene flow and enhances its suitability as a model organism for investigating the genetic impacts of both historical and contemporary barriers to dispersal.

Here we analyse the genetic variability of the Pyrenean minnow across five catchments in two basins of the Basque Country, a region where rivers are highly fragmented by both natural and artificial barriers. Our aim was to assess whether the observed patterns of genetic structure and diversity were consistent with restricted connectivity and demographic processes operating at different spatial and temporal scales, including long‐term isolation among catchments and more recent fragmentation within catchments. We tested three hypotheses: (1) we expected genetic differentiation to be stronger among catchments than among sampling sites within catchments, consistent with long‐term restrictions of gene flow among drainage systems; (2) within catchments (and after accounting for river distance), we expected sites separated by a greater number and cumulative height of barriers ≥ 2 m to exhibit stronger genetic differentiation (Prunier, Loot, et al. 2023), which would suggest an effect of contemporary dispersal restrictions on the actual genetic structure of the Pyrenean minnow; and (3) we expected the most isolated populations to exhibit lower genetic diversity and effective population size, together with stronger evidence for contemporary or historical demographic reductions.

## 2 Materials and Methods

### 2.1 Study Design

#### 2.1.1 Study Sites

The river systems of the Basque Country offer a unique opportunity to study the effects of fragmentation on minnow, as they host numerous populations within a geographically restrained region, yet displaying highly variable degrees of connectivity. This study was conducted across headwaters of five distinct catchments: Baias, Izki, Arantzazu, Agauntza and Añarbe (Figure 1). The first two belong to the Ebro River basin (Baias is a direct tributary, Izki a tributary of River Ega, itself a tributary of the Ebro), whereas the other three drain to the Atlantic Ocean, the Arantzazu as a tributary of River Deba, the Agauntza as a tributary of River Oria and the Añarbe as a tributary of River Urumea. All studied headwaters are entirely or partially within protected areas (Natura 2000 sites) and are under oceanic climate except Izki, which lies in the transition zone between the Oceanic and Mediterranean climatic regions (Table 1). All required permits for field sampling and access to study sites were obtained prior to the start of the study from the competent regional authorities in Spain (Gipuzkoa: 86‐01; Navarra: DSR_01_132E‐2022; Alava; SOL233). We studied the stream network in the uppermost part of each catchment, which ranged from 3917 to 6952 in drainage areas (Table 1).

**FIGURE 1:**
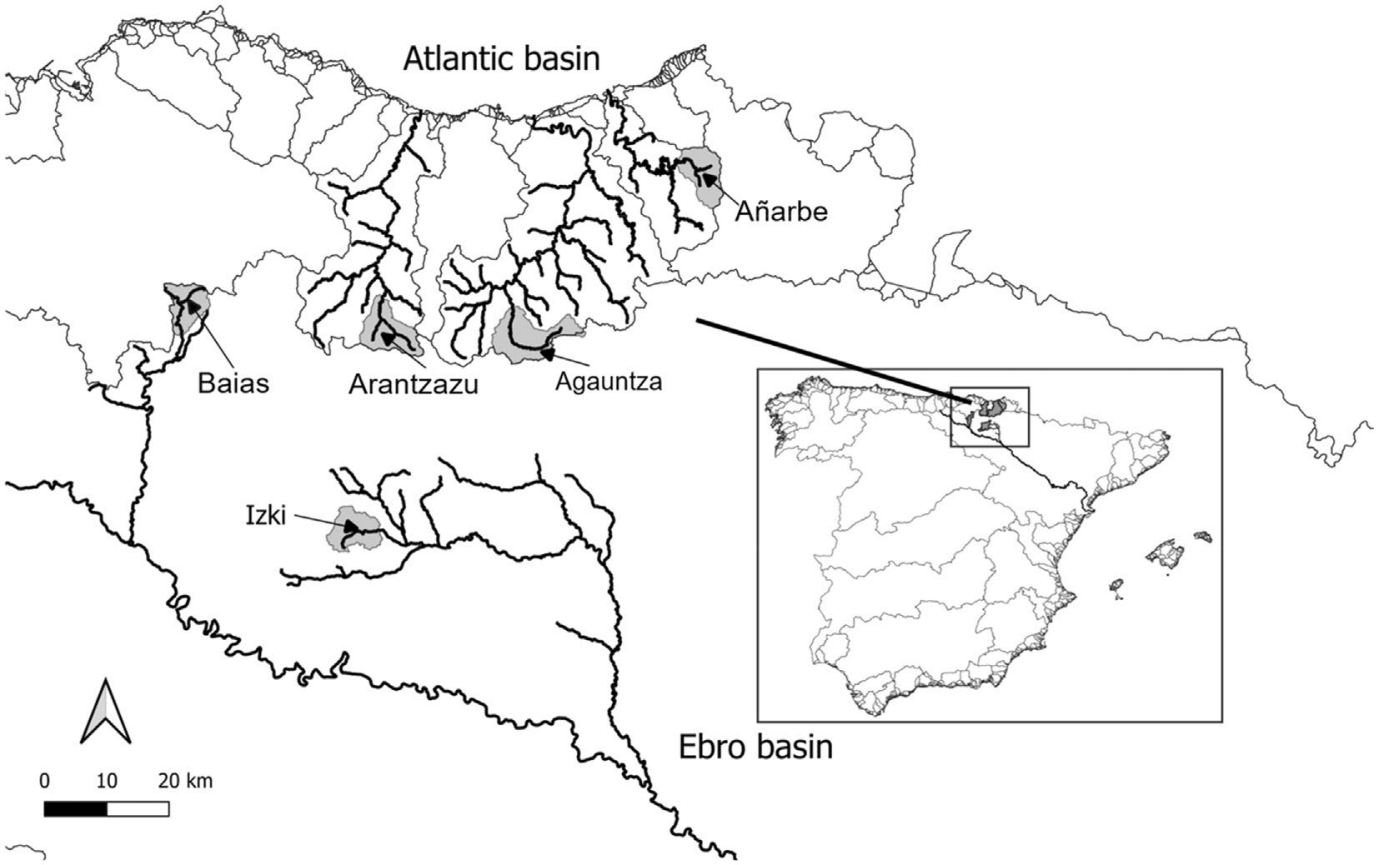
Locations of the sampled headwater catchments in the north of the Iberian Peninsula. Grey areas indicate the sections of the river network that were sampled. Catchment boundaries are delineated by grey polygons.

**TABLE 1:** Description of sampled areas.

	Baias	Izki	Arantzazu	Añarbe	Agauntza
Surface (ha)	3917	4360	5361	4709	6952
Sea	Mediterranean	Mediterranean	Atlantic	Atlantic	Atlantic
Basin	Ebro	Ebro	Deba	Urumea	Oria
Lithology	Sandstones, limestones, shales	Sandstones, limestones	Shales, sandstones, limestones	Schists, slates, granites, gravels/sands/silts	Sandstones, marls, limestones
Altitude (m)	600–800	600–800	200–800	200–400	200–500
Mean annual temperature (°C)	10.5	8.6	11.0	12.3	11.0
Mean annual precipitation (mm)	1000	870	1100	2527	1800
No. of obstacles (≥ 2 m)	25	2	22	8	33

*Note:* The surface is measured in hectares (ha), and the altitude is measured in metres above sea level (m a.s.l.).

### 2.2 Field Methods

In each catchment, we selected 10 sampling reaches (100‐m long) to provide broad spatial coverage of the stream network, resulting in a total of 50 sites surveyed. Each reach was closed with upstream and downstream nets and fish were sampled using backpack electrofishing (AGK Kronawitter, model IG200‐2). Efforts were made to collect all the fishes at those delimited areas and up to 15 minnows per site were used for the genetic analysis. Captured individuals were anaesthetised with MS‐222, measured, and a small pelvic fin clip was taken and stored in pure ethanol‐filled Eppendorf tubes. All fish were subsequently released at the site of capture. All sampling and handling of fish followed the European Directive 2010/63/EU on the protection of animals used for scientific purposes and complied with Spanish and regional regulations on animal welfare. The study was reviewed and approved by the Ethics Committee for Animal Experimentation of the University of the Basque Country (reference number: M20/2022/34). To assess the number and location of obstacles between reaches, we walked the entire river network and georeferenced all barriers, natural and artificial, and described them in terms of type (waterfall, weir, culvert, dam, dry area, etc.) and height. Only obstacles ≥ 2 m were considered for further analysis, as they have previously been shown to be potentially impassable or strongly restrictive to fish movement and, thus, alter *Phoxinus* sp. genetic connectivity (Prunier, Loot, et al. 2023). Most of these barriers were built during the late Middle Ages and the early modern period to supply water to mills and to the emerging ironworking industry, but have been often modified since, for instance, for hydropower. As the exact age of construction and of modification was unknown for most barriers, it was not considered in analyses.

### 2.3 Laboratory Methods

Fin DNA was extracted by Gentyane, the genotyping and sequencing service platform at INRAe (France), using an automatized protocol based on the DNA extraction kit DNAdvance (Beckman). Individuals were then genotyped for 48 microsatellite markers specifically developed for *Phoxinus* sp. using the SSRseq workflow described by Lepais et al. (2020); Blanchet, Prunier and Lepais, unpublished at the Plateforme Genome‐Transcriptome de Bordeaux, a high‐throughput genomics platform (INRAe, France). This workflow combines de novo primer design, multiplexed PCR amplification and high‐throughput sequencing. It increases the number of reliable loci and improves genotyping accuracy compared to traditional electrophoresis‐based methods. It also includes an automated bioinformatics pipeline to analyse sequenced data, genotyping haplotypes from complete microsatellite sequences. By targeting microsatellite loci alongside their flanking regions, SSRseq markers capture both repeat length variation and single nucleotide polymorphisms (SNPs). Consequently, the SSRseq workflow yields two kinds of allele coding: the classical allele length coding and a unique haplotypic sequence coding, with the latter resolving size homoplasy. We thus used the haplotypic sequence coding, except when stated otherwise.

### 2.4 Data Analysis

Because present‐day genetic patterns cannot be assigned unambiguously to historical or contemporary processes, we combined complementary analyses targeting different spatial and temporal components of population structure. STRUCTURE and de novo discriminant analysis of principal components (DAPC) were used at regional and within‐catchment scales to identify major genetic discontinuities, whereas pairwise $F_{\mathrm{ST}}$ and MLPE models were used to quantify genetic differentiation and test its association with present‐day river distance and barrier burden. Genetic‐diversity indices and contemporary effective population size were used to describe the current genetic and demographic condition of each population. Finally, BOTTLENECK and DIYABC‐RF were used to test for recent departures from mutation–drift equilibrium and to compare alternative demographic scenarios involving population stability or demographic reductions at different timescales respectively. Together, these analyses allowed us to evaluate whether the current patterns are consistent with processes operating over different spatial and temporal scales, although they do not provide a definitive causal partition between natural and anthropogenic fragmentation.

#### 2.4.1 Genetic Structure at Regional and Local Scales

Because the SSRseq markers used in this study were originally developed for *Phoxinus dragarum* and subsequently cross‐amplified in *P. bigerri*, we applied conservative missing‐data filters to retain a highly complete and robust marker panel. Negative controls were excluded before calculating missingness. Loci with more than 3% missing genotypes across the 285 biological samples were removed. This procedure retained 26 of the 48 initial loci; missing data ranged from 0% to 2.81% among retained loci, whereas the lowest missing‐data rate among excluded loci was 3.51%. After locus filtering, individuals with more than 10% missing genotypes across the retained loci were also excluded. The final dataset comprised 280 individuals genotyped at 26 loci, with an overall missing‐data rate of 0.38% (see Supplementary File C for further details).

To investigate the genetic structure of minnow, we used the Bayesian clustering algorithm implemented in STRUCTURE (Hubisz et al. 2009). First, all individuals across the five catchments were analysed jointly to characterise regional genetic structure and determine whether the main genetic discontinuities corresponded to drainage basins, individual catchments or groups of sites within catchments. For this analysis, the number of clusters (*K*) tested ranged from 1 to 14 (half the total number of sampling sites where we found minnows) because we expected to have different populations, but not as many as sampling sites.

To examine whether strong differentiation among catchments obscured hierarchical genetic structure or produced intermediate ancestry coefficients, we also repeated the analysis separately for the Atlantic catchments (Agauntza, Arantzazu and Añarbe) and the Mediterranean catchments (Baias and Izki).

Separate STRUCTURE analyses were conducted within each catchment to detect intra‐catchment subpopulations and to characterise finer‐scale genetic subdivision within catchments (local scale). In this case, tested *K* ranged from 1 to *n* + 1, with n being the number of sites with minnows in the corresponding catchment. Likelihood values were examined to assess the overall support for each *K* (Supplementary File A). The most likely *K* was determined using the ΔK method described by Evanno et al. (2005), which identifies the number of clusters by the largest change in likelihood (Ln*P*(*K*)) between consecutive *K* values, as implemented in STRUCTURE HARVESTER (Earl and vonHoldt 2012). To align and summarise results across replicates, CLUMPP (Jakobsson and Rosenberg 2007) was used with the Greedy algorithm. The lack of structure at *K* = 2 was interpreted as being *K* = 1 (no genetic structure).

As a complementary multivariate analysis of genetic structure, we performed de novo discriminant analyses of principal components (DAPC) using the adegenet package in R (Jombart 2008). The same final dataset of 280 individuals genotyped at 26 SSRseq loci was used, and missing genotypes were replaced by the mean allele frequency within each analysed subset; the overall missing‐data rate was 0.38%. Genetic clusters were inferred without prior information on catchment or sampling‐site identity using successive *K*‐means clustering implemented in find.clusters. Analyses were conducted for the complete regional dataset, separately for the Atlantic and Mediterranean drainages, and independently within each catchment. For the clustering step, we retained the number of principal components required to represent 90% of the total genetic variance. The number of clusters was selected by inspecting the Bayesian information criterion (BIC) profile and retaining the smallest *K* after which additional clusters produced no appreciable improvement in model fit; *K* = 1 was retained when increasing *K* did not reduce the BIC. For each selected *K*, the subsequent DAPC retained *K* − 1 principal components and up to *K* − 1 discriminant functions, following Thia (2022). Catchment and sampling‐site identities were used only after clustering to evaluate the geographical composition of the groups inferred de novo.

#### 2.4.2 Landscape Correlates of Within‐Catchment Differentiation

To test whether within‐catchment genetic differentiation was associated with present‐day network configuration and barrier burden, we employed a Maximum Likelihood Population Effects (MLPE) model (Clarke et al. 2002) following the pipeline provided by Dalapicolla (https://github.com/jdalapicolla/MLPE.R/blob/master) in RStudio (RStudio Team 2020). This statistical framework is an alternative to partial Mantel tests (or regression on distance matrices) that fits linear mixed models to pairwise data, incorporating random effects to account for the non‐independence among comparisons. In our model, the response variable was genetic distance—calculated as Nei's *F*_ST_ index (Nei 1987)—and we used catchment identity as an additional hierarchical random effect. The fixed effects included river distance, cumulative obstacle height, number of obstacles between sites and water‐quality distance. Water‐quality distance was calculated from pH, conductivity, water temperature, oxygen saturation, chloride, sulphate, nitrate, ammonium and phosphate. Euclidean distances were then calculated between each pair of sampling sites. All the combinations of these explanatory variables were evaluated to identify the best‐fitting model explaining genetic differentiation.

#### 2.4.3 Contemporary Genetic Diversity and Effective Population Size

To characterise the contemporary genetic and demographic condition of the populations, we calculated basic population genetic statistics, including observed (Ho) and expected heterozygosity (He) as well as allelic richness (Ar) using the hierfstat package (Goudet 2005) implemented in RStudio (RStudio Team 2020). We also estimated contemporary Ne using the NeEstimator v2.1 software (Do et al. 2014), applying the linkage disequilibrium method (Hill 1981). A minimum allele frequency threshold of 0.02 was used to reduce upward bias in Ne estimation due to rare alleles. Since strong population structure can bias Ne estimates when using the linkage disequilibrium method—typically leading to underestimation—all the statistics were estimated for each full catchment, and then for each subpopulation within a catchment, providing there were enough samples (> 5). However, these subpopulations sometimes had very few individuals, so results should be interpreted with caution. Allelic richness values are comparable only within the same hierarchical level (populations on one hand, and subpopulations within populations on the other hand, separately), since rarefaction is standardised to the smallest sample size within each dataset. Although rarefaction standardises allelic‐richness estimates for differences in sampling effort, it does not eliminate uncertainty in allele‐frequency estimates or the reduced probability of detecting low‐frequency alleles in the smallest samples (Hale et al. 2012).

#### 2.4.4 Recent Demographic Changes and Alternative Demographic Histories

Additionally, we analysed the series of past events leading to the current genetic structure of the species in the region. We first ran the BOTTLENECK software (Cornuet and Luikart 1996) using SSRseq allele length coding rather than haplotypic coding, since BOTTLENECK analyses rely on allele size information under mutation models such as two‐phase mutation model (TPM) and stepwise mutation model (SMM). Analyses were performed under TPM, with 90% of mutations assumed to follow the SMM and a variance of the geometric distribution set at 10 (Paz‐Vinas et al. 2013). The results were interpreted considering the Wilcoxon test, because it is the most trustworthy test regarding the scarcity of samples at some sites (Luikart et al. 1998). Then, considering those results, we used DIYABC‐RF software (Collin et al. 2021) to refine scenarios and estimate demographic parameters such as the number of generations since population variation and the effective population sizes before and after a particular event. Because DIYABC‐RF requires allele size information for microsatellite‐based analyses, we here used microsatellite allele length coding rather than haplotypic sequence coding. This software uses an Approximate Bayesian Computation (ABC) approach and combines simulation‐based modelling with random forest machine learning to identify the scenario best fitting the observed genetic data. For each catchment, we independently tested four competing demographic scenarios, which were produced using both a priori information (see below) and a posteriori according to main trends observed in the datasets (based on Ne, He, AR, etc., see Section 3): Sc1: A historically large stable population with no demographic change over time; Sc2: a historically large population that experienced a recent or post‐medieval bottleneck (10–600 years); Sc3: a historically large population that experienced an older bottleneck between 1000 and 7500 generations ago, encompassing late‐Pleistocene and Holocene demographic changes; and Sc 4: A historically small population that remained demographically stable. The scenarios were simulated 395,000 times per catchment, with the simulations divided equally among the four scenarios. Priors were defined using broad uniform distributions to capture biologically realistic ranges. Assuming a 2‐year generation time for minnows, the time since post‐medieval bottlenecks was set between 5 and 300 generations (i.e., 10–600 years, timeframe at which these catchments were hugely impacted by humans after the medieval ages; Almeida et al. 2023), while the time since ancient bottlenecks was set between 1000 and 7500 generations (i.e., 2000–15,000 years), encompassing the Late Glacial period, including the Younger Dryas, and the subsequent Holocene, climatic periods that have left strong signatures on the genetic structure of European freshwater fishes (April et al. 2013; Wilson and Bernatchez 1998). Effective population sizes before the bottleneck were drawn from a uniform distribution between 1000 and 10,000 individuals, and post‐bottleneck sizes ranged from 5 to 300 individuals, based a posteriori on the results. Prior‐ scenario compatibility was assessed using the prior‐scenario checking procedure implemented in DIYABC‐RF, by projecting the observed datasets onto a principal component analysis of datasets simulated under the four demographic scenarios (Figure S3).

## 3 Results

### 3.1 Sampling Coverage and Barrier Characteristics

In total, we captured 1430 *Phoxinus bigerri* at 28 sampling sites, of which 285 individuals were selected for genotyping and 280 were retained after missing‐data filtering. The final dataset comprised 20 individuals from Arantzazu, 61 from Agauntza, 43 from Añarbe, 124 from Baias and 32 from Izki, and the number of sampled sites where fish were found ranged from 3 (Izki) to 10 (Baias) (Table S1). Barrier number and cumulative height varied considerably among catchments, ranging from no barrier in Izki between sites where samples were found to 25 barriers in Baias, eight of which were artificial. The relative contribution of artificial barriers also differed among catchments, representing between 14% and 67% of the total number of barriers and between 23% and 80% of their cumulative height in catchments where artificial barriers were present (Table S2). However, natural and artificial barriers were considered together in these analyses; thus, the models quantified the overall barrier burden between sampling sites but did not separately estimate the effects of barrier origin. Separate effects were not fitted because the available number and distribution of barriers did not allow a robust comparison between barrier types, and reliable information on barrier age was unavailable for many structures.

### 3.2 Regional and Local Genetic Structure

At a regional scale, STRUCTURE analysis (Figure 2) revealed clear genetically differentiated populations among catchments, with the best *K* being 5 based on ΔK values (Supplementary File A). Within the Atlantic‐draining basins, individuals from Añarbe, Arantzazu and Agauntza were predominantly assigned to distinct genetic clusters, a pattern consistent with limited gene flow among catchments. Individuals from Izki, located within the Mediterranean‐draining Ebro basin, also exhibited a distinct genetic composition and were clearly differentiated from those sampled in the Atlantic basins. Baias showed a more complex assignment pattern: approximately half of the individuals were predominantly assigned to a Baias‐associated cluster, whereas the remaining individuals displayed mixed membership between this cluster and the cluster predominant in Izki. Overall, the analysis suggested five main genetic clusters broadly associated with catchments, with intermediate membership coefficients occurring mainly in Baias relative to the Izki‐associated cluster.

**FIGURE 2:**
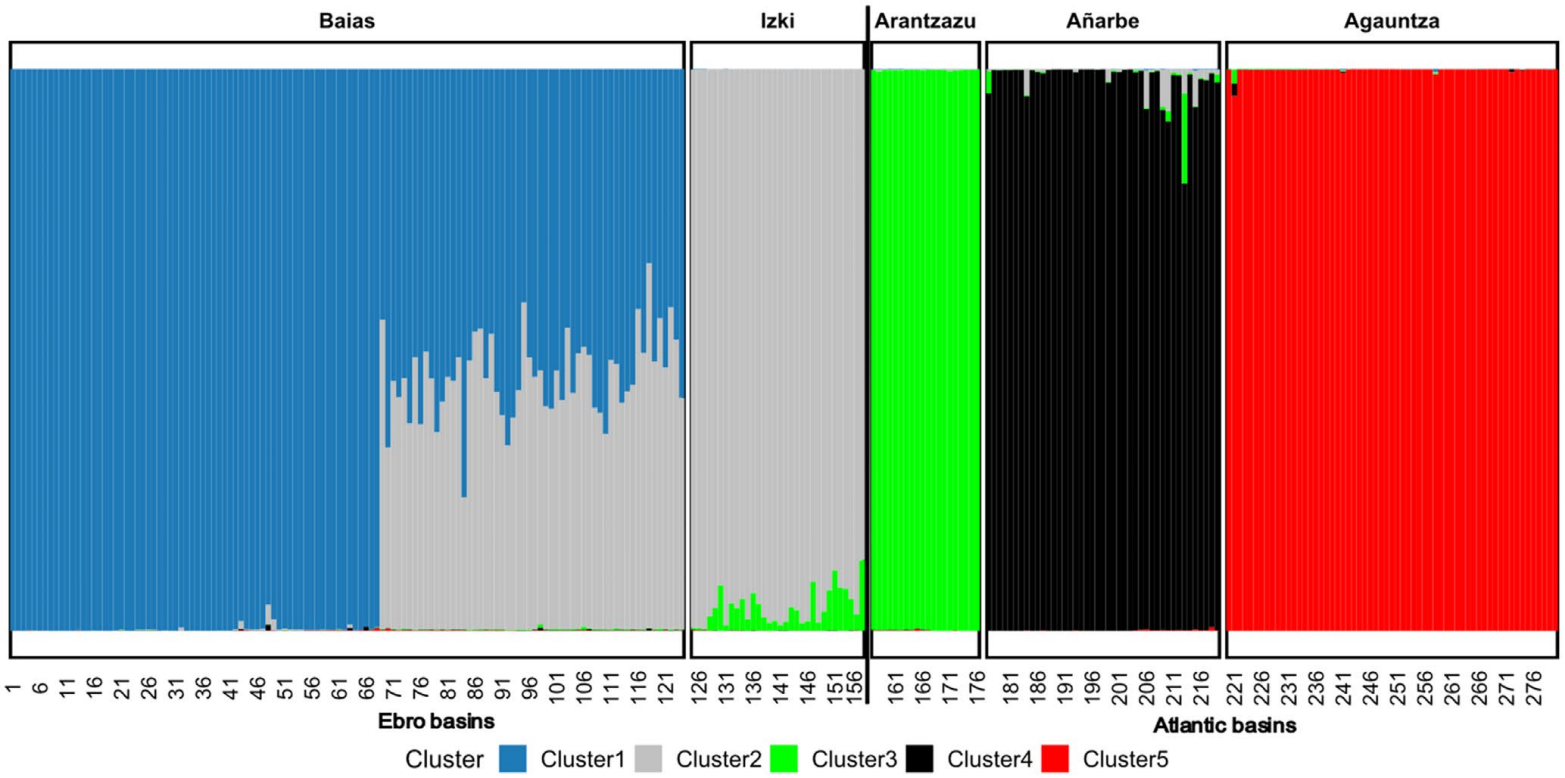
Genetic structure of minnow individuals across the five sampled catchments, as inferred by STRUCTURE analysis (*K* = 5). Each vertical bar represents an individual, and the colours indicate the estimated proportion of ancestry assigned to each of the five genetic clusters. The thick black vertical line separates individuals from the two different basins. The *x*‐axis corresponds to individual sample IDs (ordered by sampling sites), with each vertical bar representing an individual and the colour segments indicating the estimated ancestry proportion. Clusters generally corresponded to their geographical locations, except for Baias, where individuals exhibited a clear admixture between two clusters.

The separate drainage‐level analyses recovered strong differentiation among catchments. In the Atlantic subset, *K* = 3 predominantly assigned individuals according to their catchment of origin, with Agauntza, Arantzazu and Añarbe corresponding to distinct genetic clusters. The analysis of the Mediterranean subset recovered the broad differentiation between Baias and Izki observed in the complete dataset (Supplementary File A).

To characterise fine‐scale population structure of minnow and its potential association with within‐catchment fragmentation, we analysed each catchment independently. At a local scale, no substructure was detected in Izki and in Agauntza (Figure 3). In contrast, both Arantzazu and Baias exhibited two distinct subpopulations. In Arantzazu, minnows from Site 3 formed apresented one subpopulation clearly differentiated from those at the other three sites where minnows were present. In Baias, individuals upstream from Site 6 were clearly genetically distinct from the ones downstream, confirming previous findings at the regional scale (Figure 2). Añarbe also showed two distinct subpopulations but a more complex pattern, with a fully admixed group primarily confined to the main stream (Sites 5 and 10) and two more differentiated subpopulations at the tributaries (Sampling Site 6 on one hand, and Sampling Sites 4 and 1 on the other hand). However, the within‐catchment STRUCTURE analysis revealed mixed cluster membership among individuals from different areas (Supplementary File A), suggesting incomplete genetic differentiation among the inferred groups.

**FIGURE 3:**
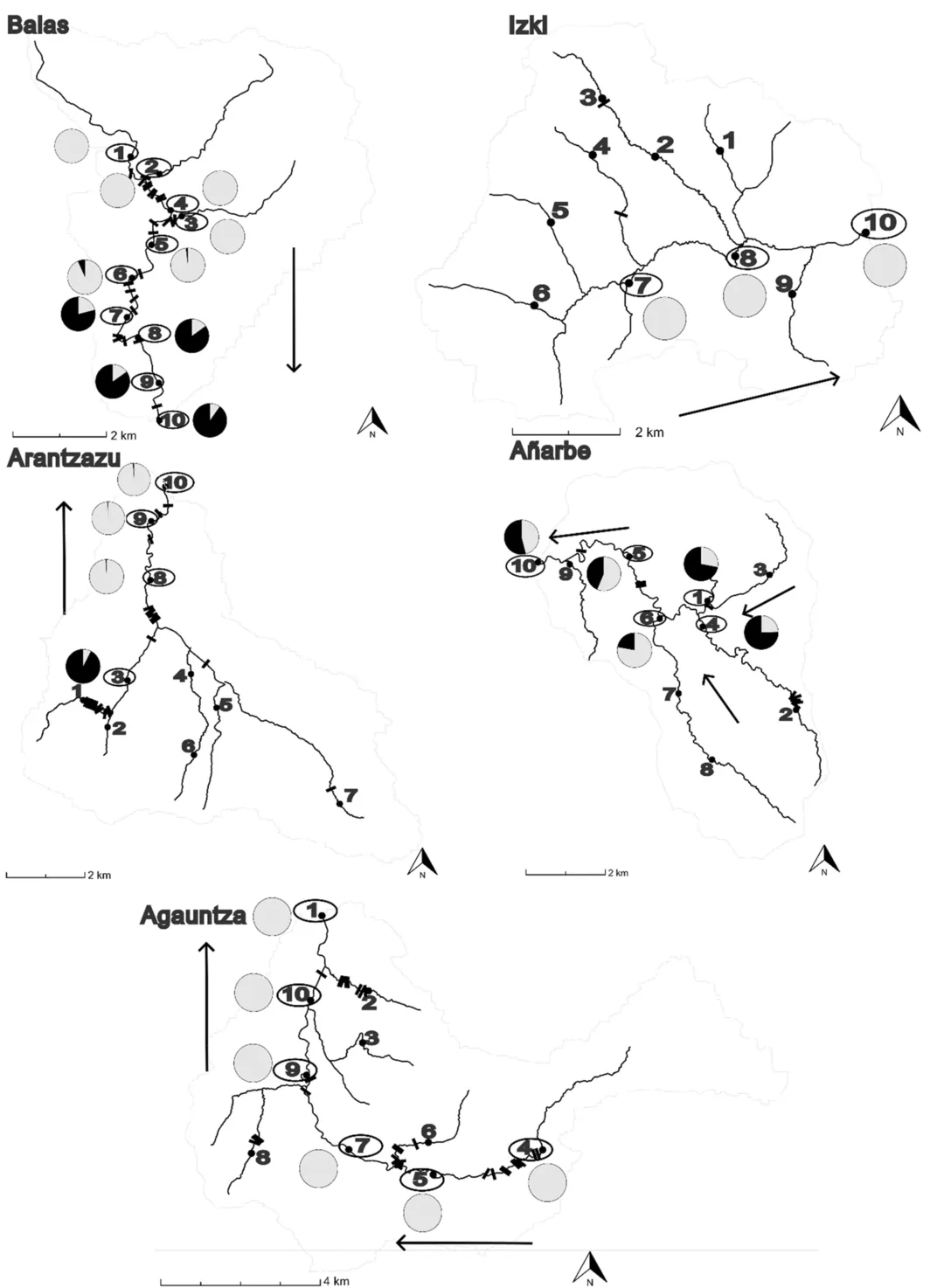
Maps of the river networks in the Baias, Izki, Arantzazu, Añarbe and Agauntza study areas. Sampling sites are indicated by dots, and fluvial obstacles exceeding 2 m in height are shown as perpendicular lines. Sites where minnows were present are circled. The numbers displayed next to the sampling sites correspond to the site identifiers used in the Supplementary Material, where the number of individuals sampled at each site is also provided. The genetic substructure of minnow populations is represented next to each sampling site. Pie charts show the mean ancestry coefficients of individuals at each site based on Bayesian clustering analyses performed in STRUCTURE. The optimal number of genetic clusters was *K* = 1 for Izki and Agauntza and *K* = 2 for Baias, Añarbe and Arantzazu (see Supplementary File A for details).

The de novo *K*‐means and DAPC analyses broadly corroborated the spatial patterns identified by STRUCTURE (Figure 4; Figures S1 and S2). At the regional scale, the BIC profile supported six genetic clusters. Individuals from Arantzazu, Agauntza, Añarbe and Izki were assigned to separate catchment‐specific clusters, whereas Baias was divided into two additional groups corresponding almost completely to Sites 1–6 and Sites 7–10. Thus, the additional cluster relative to the regional STRUCTURE result reflected the within‐Baias subdivision rather than an additional catchment‐level lineage. The drainage‐specific analyses recovered three clusters in the Atlantic drainage, corresponding exactly to Arantzazu, Agauntza and Añarbe, and three clusters in the Mediterranean drainage, corresponding to Izki and the two Baias groups. Within catchments, DAPC recovered the separation of Arantzazu Site 3 from Sites 8–10 and the division of Baias between Sites 1–6 and 7–10, with only one individual from Baias Site 8 assigned to the opposite group. In Añarbe, the three inferred clusters were distributed across multiple sampling sites and did not show a clear spatial correspondence. Similarly, the two groups inferred in Agauntza were interspersed among sites, whereas the BIC supported a single cluster in Izki. These results therefore supported strong regional differentiation and the pronounced spatial subdivisions identified in Arantzazu and Baias, while providing no evidence of discrete spatial structure in Agauntza or Izki and confirming the more complex pattern observed in Añarbe.

**FIGURE 4:**
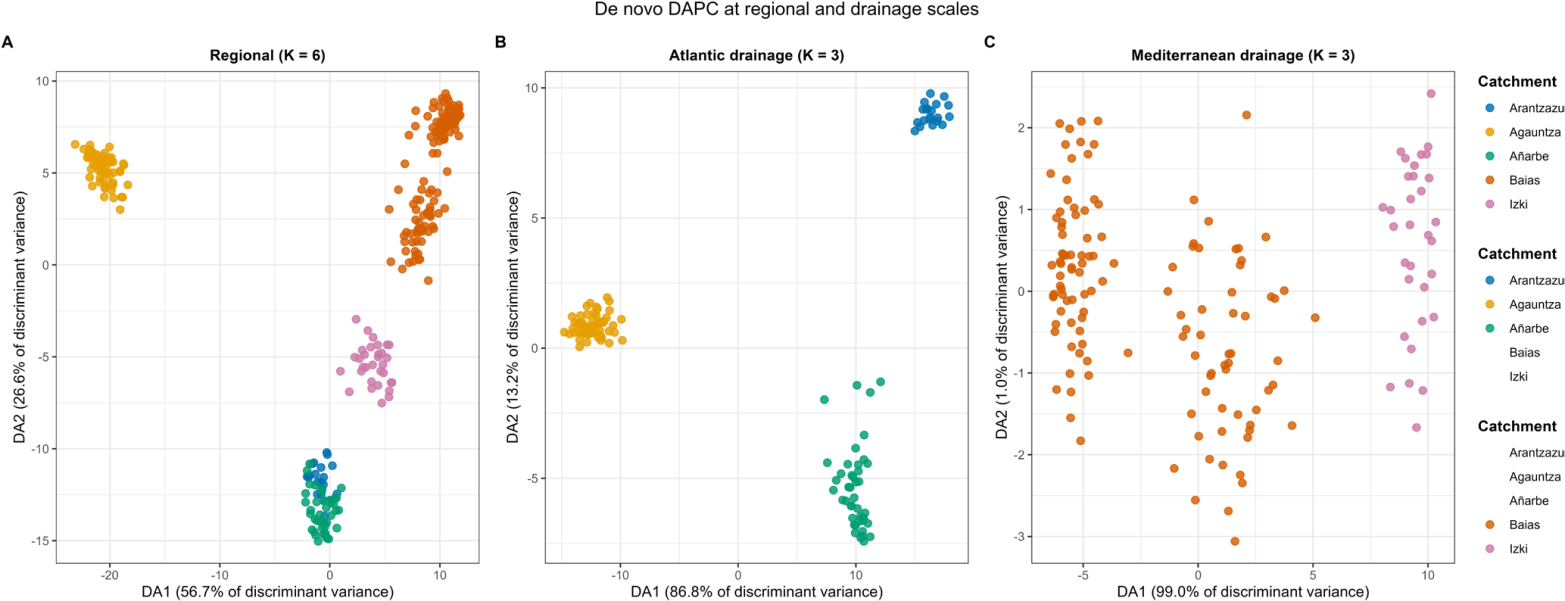
De novo discriminant analyses of principal components (DAPC) for (A) the complete regional dataset, (B) the Atlantic drainage and (C) the Mediterranean drainage. Individuals are coloured according to catchment of origin, which was used only after de novo clustering to describe the geographical composition of the inferred genetic groups.

### 3.3 Landscape Correlates of Within‐Catchment Differentiation

The MLPE analysis identified the number of obstacles ≥ 2 m as the strongest predictor of genetic distance within catchments (Table 2). The best‐supported model included only the number of intervening obstacles, whereas a second model additionally including water‐quality distance received slightly less support (ΔAIC = 1.22). Water‐quality distance showed no detectable additional association with genetic differentiation (estimate = −0.006 ± 0.007, *p* = 0.384), and the estimated effect of barrier number remained virtually unchanged after accounting for physicochemical differences among sites. Neither river distance nor cumulative obstacle height received comparable model support. Genetic differentiation increased with the number of obstacles (estimate = 0.068 ± 0.007, *p* < 0.001; Figure 5).

**TABLE 2:** Model selection results from MLPE analyses evaluating the effects of the number of obstacles (N_obstacles), cumulative obstacle height (AcumHeight), river distance (RiverDistance), pairwise water‐quality distance (Water_quality) on genetic distances between populations. Bold values indicate the best‐supported model according to AIC. The effect of the number of obstacles was statistically significant (estimate = 0.068, SE = 0.00695, *p* < 0.001).

Intercept	N_obstacles	AcumHeight	RiverDistance	Water_quality	DF	LogLik	AIC	Delta	Weight
**0.120**	**0.068**				**5**	**118.768**	**−227.535**	**0.00**	**0.648**
0.121	0.069			−0.006	6	119.159	−226.317	1.22	0.352
0.109		0.064			5	107.613	−205.2	22.31	< 0.001
0.112		0.065		−0.010	6	108.415	−204.8	22.70	< 0.001
0.094			0.034		5	96.988	−184.0	43.56	< 0.001
0.097			0.037	−0.008	6	97.398	−182.8	44.74	< 0.001
0.100					4	90.012	−172.0	55.51	< 0.001
0.099				0.002	5	90.033	−170.1	57.47	< 0.001

*Note:* Estimated intercept and predictor coefficients, degrees of freedom (DF), log‐likelihood (logLik), Akaike Information Criterion (AIC), delta AIC (difference from the most supported model) and Akaike weights for the best‐fitting models. Lower AIC and higher weight indicate higher model support. The best‐fitting model included only the number of obstacles, highlighting its significant effect on genetic structure.

**FIGURE 5:**
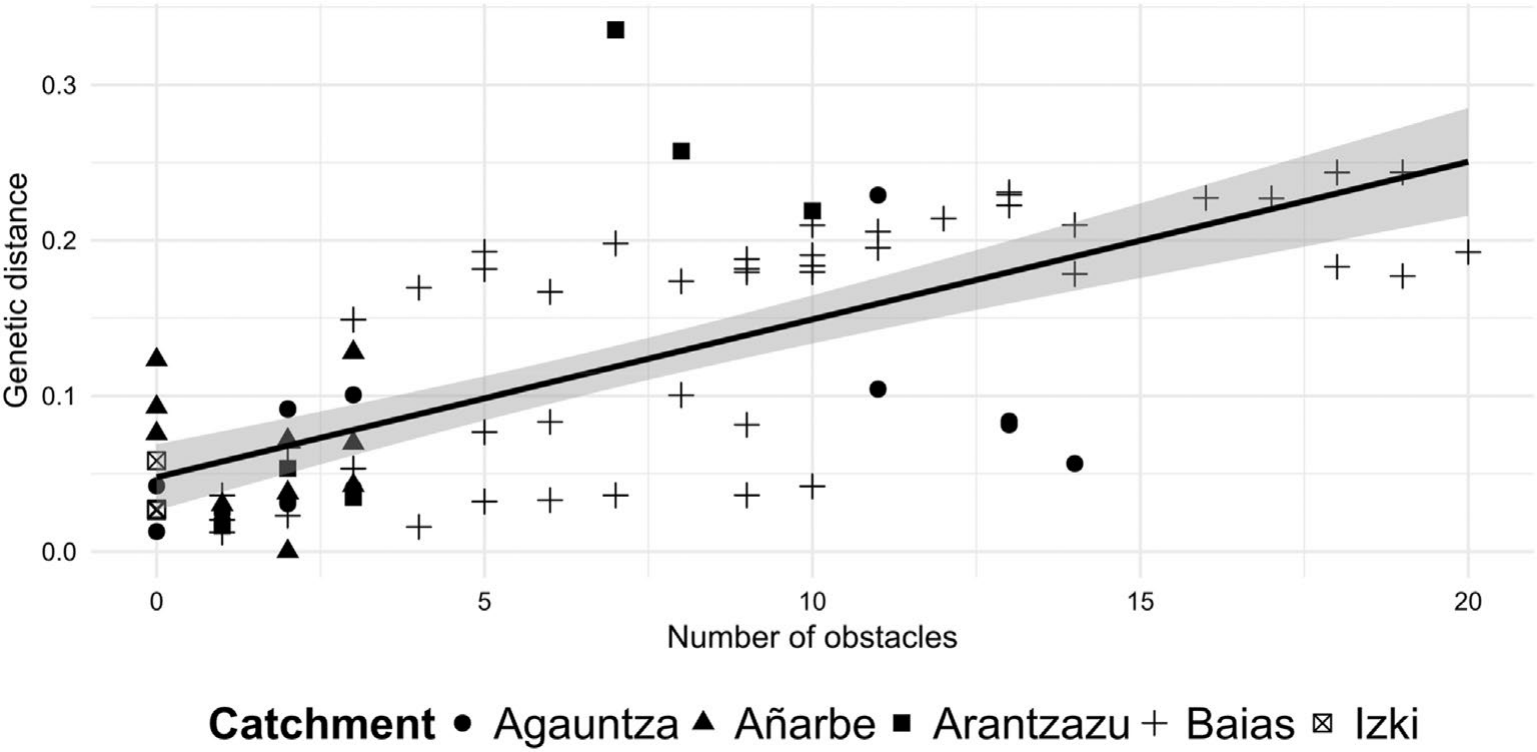
Relationship between the number of river obstacles (*x*‐axis) and genetic distance of minnows (*y*‐axis) among site pairs within catchments. Each symbol represents a pairwise comparison between sampling sites within each catchment, with different shapes corresponding to different catchments (Arantzazu, Agauntza, Añarbe, Baias and Izki). The black line represents the fitted linear regression, and the shaded area shows the 95% confidence interval.

### 3.4 Contemporary Genetic Diversity and Effective Population Size

Consistent with this pattern of strong genetic differentiation, both Ho and mean Ar were generally low, particularly in Arantzazu (Ar = 2.654; Ho = 0.326) (Table 3). In Baias, the difference between the two subpopulations was especially pronounced: the isolated sites upstream from Site 6 showed lower Ho and Ar (Upstream: Ho = 0.231, Ar = 1.909) and lower Ne (minimum Ne = 133.2) than any downstream sites (Ho = 0.587, Ar = 3.839, minimum Ne = 250.3). This contrast between the catchment‐level values (Table 3) and the subpopulation‐specific estimates shows that pooling all individuals from the catchment inflates the overall diversity estimates, because it ignores the strong genetic substructure within the catchment. Consistently, estimates of Ne were also low in most catchments, especially in the Atlantic basins, where the lower bounds of the confidence intervals were notably small (Arantzazu: 29; Añarbe: 19.1; Agauntza: 196.2) (Table 3). Although confidence intervals were broad, the consistently low lower confidence bounds—particularly in Arantzazu and Añarbe—further support the evidence of reduced genetic variation, small Ne and potential demographic constraints.

**TABLE 3:** Mean values and standard deviation (SD) of Ar, Ho and He, and estimated 95% confidence intervals for Ne for each population and subpopulation in the sampled catchments.

Catchment	Group	Ar	Ho	He	Ne
Baias	Whole catchment	4.068 (1.31)	0.392 (0.23)	0.402 (0.21)	9–10.4
	Sites 1–6	1.909 (0.88)	0.231 (0.26)	0.204 (0.21)	133.2–∞
	Sites 7–10	3.839 (1.16)	0.587 (0.24)	0.543 (0.23)	250.3–∞
Izki	Whole catchment	4.446 (1.76)	0.581 (0.21)	0.592 (0.21)	105.7–756.8
Arantzazu	Whole catchment	2.654 (1.23)	0.326 (0.26)	0.300 (0.23)	24.9–∞
	Sites 7–8–9	2.276 (1.06)	0.342 (0.26)	0.296 (0.23)	40.6–∞
Añarbe	Whole catchment	4.666 (2.20)	0.445 (0.25)	0.493 (0.24)	19.1–24.2
	Tributaries	3.729 (1.52)	0.433 (0.24)	0.486 (0.23)	5.2–7.3
	Main stream	4.154 (2.33)	0.486 (0.32)	0.476 (0.28)	57.9–∞
Agauntza	Whole catchment	3.592 (2.06)	0.437 (0.29)	0.420 (0.29)	196.2–∞

*Note:* Results from Añarbe, Arantzazu and Baias are also shown for subpopulations because substructure was detected at those sites. The strong differences between population estimates and subpopulation estimates for Baias indicated that some population estimates were probably biased by genetic substructure.

### 3.5 Recent Demographic Changes and Alternative Demographic Histories

Despite the low Ne estimates, BOTTLENECK provided no evidence of a widespread recent bottleneck across catchments, although Izki showed significant heterozygosity excess under the TPM. Under this model, the Wilcoxon test did not detect significant heterozygosity excess in Arantzazu (*p* = 0.892), Agauntza (*p* = 0.931), Añarbe (*p* = 0.932) or either of the two Baias subpopulations, indicating no clear signal of a recent bottleneck in these cases. In Baias, individuals were analysed as two separate subpopulations, corresponding to Sampling Sites 1–6 (Cluster 1) and 7–10 (Cluster 2), because the previous STRUCTURE analysis revealed a genetic subdivision between these two groups, with enough samples to perform the following analyses. The Baias subpopulation including Sites 1–6 showed significant heterozygosity deficiency under TPM in the one‐tailed Wilcoxon test (*p* = 0.041), suggesting a pattern more consistent with recent expansion or demographic recovery than with a contemporary reduction in population size. In contrast, the Baias subpopulation including Sites 7–10 showed no significant evidence of heterozygosity excess or deficiency under TPM. Izki was the only population showing significant heterozygosity excess under the one‐tailed Wilcoxon test under TPM (*p* = 0.028). Overall, these results suggest that the observed low genetic diversity and low Ne are unlikely to reflect a widespread very recent bottleneck detectable through transient heterozygosity excess, but may instead be associated with older demographic processes, long‐term fragmentation or population‐specific demographic histories.

To further investigate the demographic history underlying the observed genetic patterns, we tested four alternative scenarios using DIYABC‐RF. As shown in Figure 6, DIYABC‐RF did not support a single demographic scenario across all populations. However, the post‐medieval bottleneck scenario (Sc2) was selected in four cases: sites in Baias downstream from Sampling Site 7 (Cluster 2), Izki, Arantzazu and Agauntza. Support for this scenario was particularly strong in Izki, where Sc2 received almost all random forest votes and a very high posterior probability (posterior probability = 0.991), and in Arantzazu (posterior probability = 0.833). Cluster 2 in Baias also supported Sc2, although with more moderate confidence (posterior probability = 0.625), while Agauntza showed weaker support for Sc2 (224 vs. 194; posterior probability = 0.653). In contrast, Añarbe strongly supported Sc1, corresponding to a large population with no bottleneck history (660 votes; posterior probability = 0.935). Cluster 1 in Baias selected Sc4, representing a population that has remained small over time, but this result should be interpreted cautiously given its low posterior probability (0.323). Overall, the post‐medieval bottleneck scenario received strongest support in several populations, particularly Izki and Arantzazu, whereas other populations supported alternative demographic histories or showed greater uncertainty among scenarios. Parameter estimates of effective population sizes before and after demographic change, together with the estimated timing of the inferred bottleneck events, are shown in Figure 7. Prior‐scenario checking showed that the observed datasets fell within the multivariate space generated by the simulations in all analyses, with the first two components explaining approximately 94% of the total variation (Figure S3).

**FIGURE 6:**
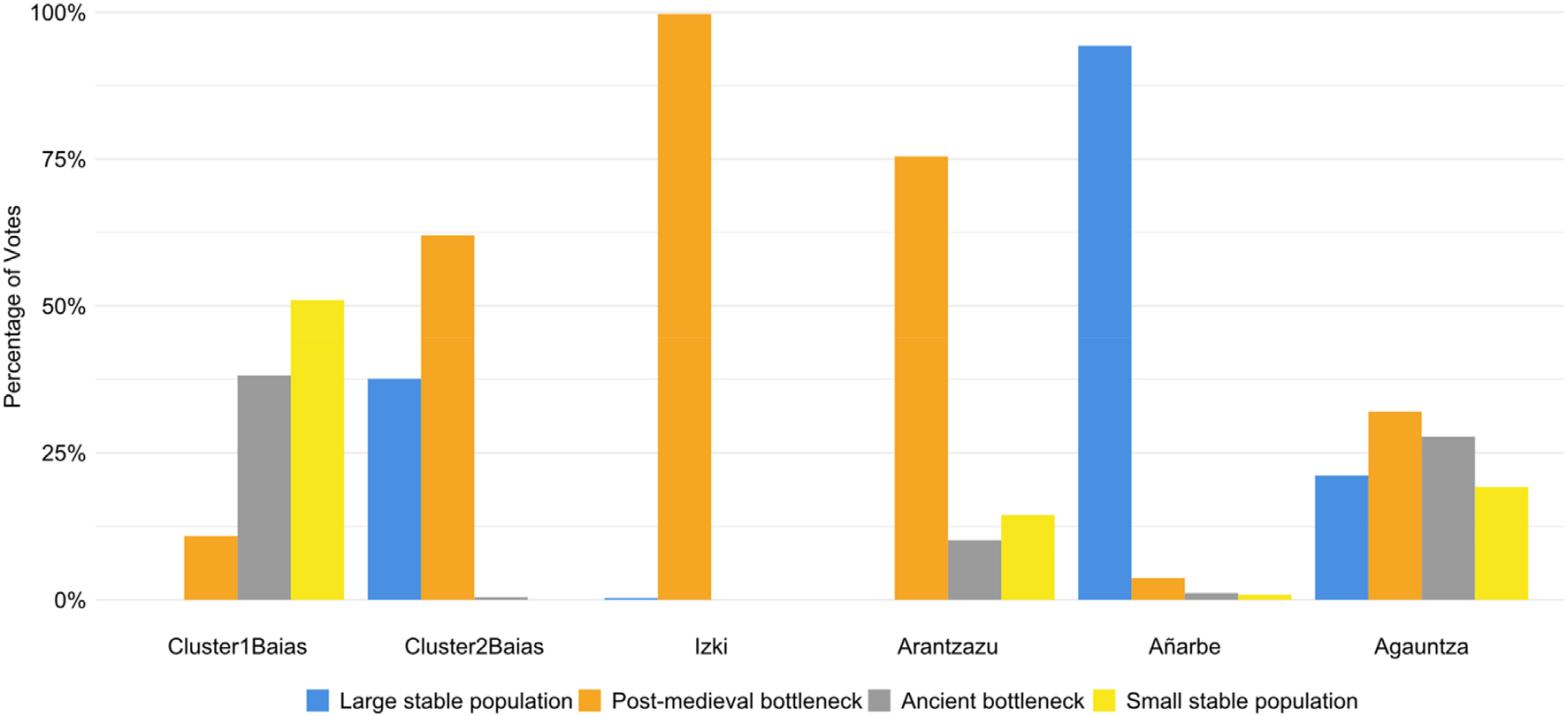
Proportion of votes received by each a priori scenario in the DIYABC‐RF analysis, based on 700 trained decision trees. Populations at most of the catchments strongly supported the scenario of a post‐medieval bottleneck, likely of anthropogenic origin. Añarbe supported the scenario of a large stable population without bottleneck, while the subpopulation at the upstream part of Baias presented a small stable population.

**FIGURE 7:**
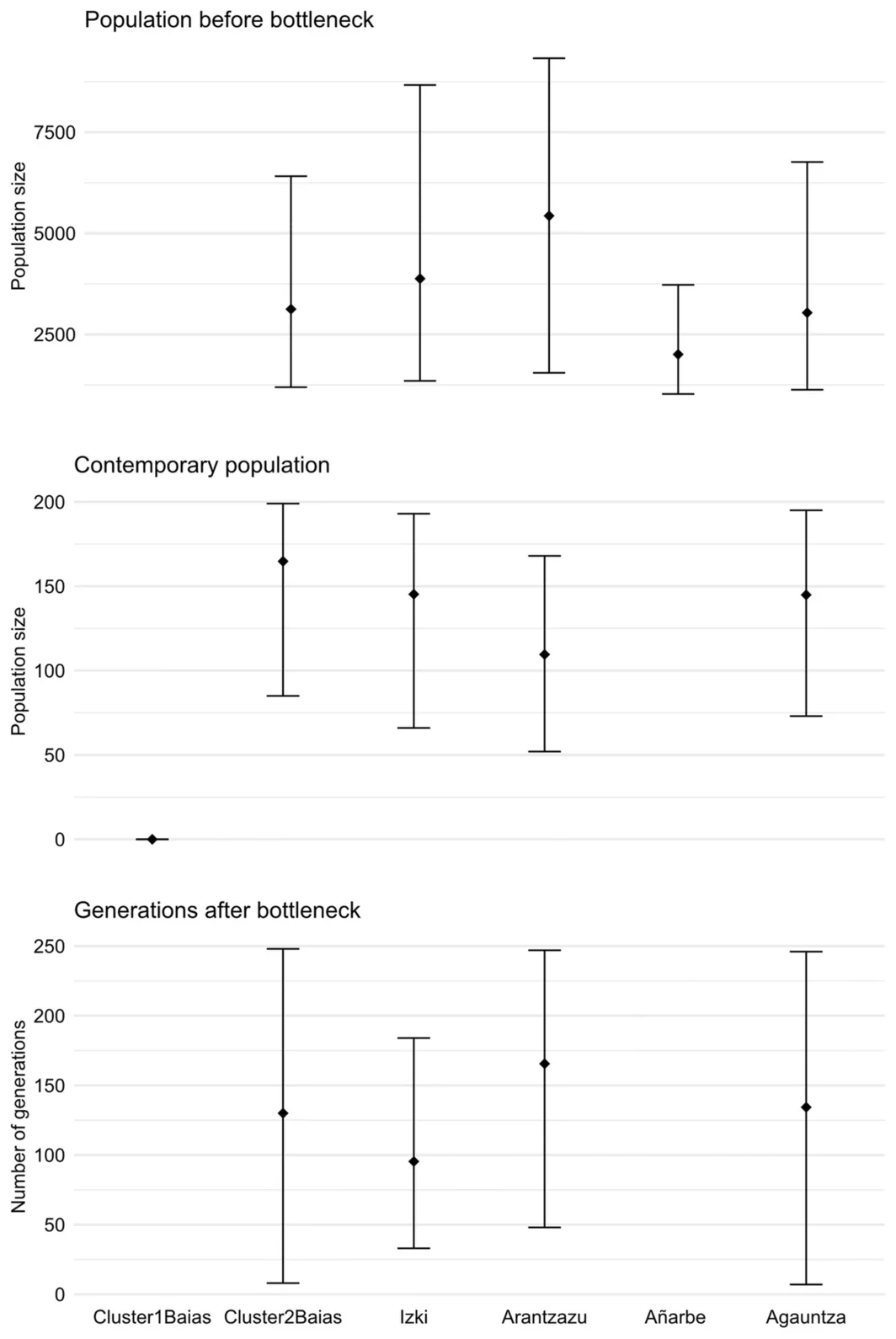
Parameter estimates obtained using the DIYABC‐RF software. Panels show (from top to bottom): Estimated population size before the bottleneck, contemporary population size and the number of generations since the bottleneck event. Dots represent the posterior mean estimates obtained via Random Forest regression, and vertical lines indicate the 5th and 95th percentiles of the posterior distribution. No estimate is available for Cluster1Baias in the ‘Population before bottleneck’ panel because the most probable scenario, despite its low posterior probability, was the ‘Small stable population’ scenario, which does not include a bottleneck event. Similarly, the estimate for ‘Population after bottleneck’ is close to zero, but it should be interpreted as indicating a very small effective population size rather than a true value of zero, given the limited reliability of the scenario choice (posterior probability = 0.323). No estimate is available for Añarbe in the ‘Population after bottleneck’ panel because the most probable scenario was the ‘Large stable population’ scenario, which also does not include a bottleneck. Consequently, these two groups do not have estimates for the number of generations since the bottleneck event.

## 4 Discussion

Genetic patterns in many freshwater species reflect a complex overlay of ancient natural isolation and more recent human‐induced fragmentation (Storfer et al. 2010). A key question is whether the effects of these recent barriers can be detected, or whether genetic patterns remain dominated by ancient processes. In our dataset, strong genetic differentiation among catchments is consistent with long‐term isolation potentially predating the Last Glaciation, whereas finer‐scale analyses revealed additional patterns of differentiation within catchments associated with present‐day fragmentation.

In our dataset, population isolation among catchments traces back to processes predating the Last Glaciation and largely explains the regional genetic signature of the studied species, whereas finer‐scale analyses revealed additional effects of more recent, likely post‐medieval, habitat changes caused by human activities. Beyond the five distinct populations that were identified—one per catchment, except for the two Mediterranean catchments that shared an admixed population—genetic distance within catchments increased with the number of barriers between sites. This pattern indicates that even moderate within‐catchment fragmentation can restrict dispersal and promote genetic drift. Demographic reconstructions further suggested bottlenecks occurring approximately 160–430 years ago (Figure 7), consistent with a period of intensified river modification in the region. The pronounced genetic differentiation among catchments at a regional scale is in line with previous studies on minnow, indicating limited gene flow among catchments, consistent with long‐term isolation imposed by basin boundaries (Corral‐Lou et al. 2019). Despite the geographic proximity of our catchments (30–90 km), genetic differentiation was apparent. The influence of ancient glacial–interglacial cycles on genetic structuring has been demonstrated in other *Phoxinus* species (Artaev et al. 2025). The sampled catchments, particularly those on the Atlantic side, seem to have remained isolated since at least the end of the Pleistocene (Corral‐Lou et al. 2019). During the Last Glacial Maximum (≈15,000–20,000 years ago), substantially lower sea level (Cearreta 1994; García‐Artola et al. 2023) extended river courses across the continental shelf. Where adjacent palaeochannels converged, this could have temporarily increased connectivity among currently isolated catchments, as proposed for other Iberian freshwater fishes (Filipe et al. 2009; Perea et al. 2021). However, lower sea level alone does not demonstrate that the focal catchments were hydrologically connected, and no detailed palaeodrainage reconstruction is available for the study area. Furthermore, Pleistocene river diversions and reconfiguration of catchments seem to have been common in the Basque region (Iribar and Ábalos 2023), making it difficult to reconstruct the history of potential connections among fish populations. Post‐glacial sea‐level rise in the early Holocene likely re‐established the present hydrological configuration, isolating catchments again and probably reducing any opportunities for coastal connectivity, contributing to the present isolation among catchments (Cearreta 1994; García‐Artola et al. 2023). Yet, our genetic data do not point to a dominant role of that transient glacial connectivity. Instead, isolation may have persisted because the relatively low discharge of these catchments was insufficient to establish effective downstream connections across the exposed continental shelf during glacial low‐sea‐level periods, as previously proposed for the Artibai Basin (Corral‐Lou et al. 2019), and, consequently, the genetic signature of the basin isolations might predate the Last Glaciation. Conversely, our findings suggest that sites within the Ebro basin may have remained partially connected in the past, as indicated by the admixture observed in populations from Baias. An alternative but unlikely explanation for this pattern would be human‐mediated translocation of minnows between these two catchments, perhaps by live‐baiting anglers.

While ancient processes largely explain the regional genetic structure, two methodologically distinct clustering approaches provided broadly congruent evidence of strong differentiation among catchments. STRUCTURE and de novo DAPC both separated the three Atlantic catchments, while the DAPC additionally recovered the pronounced subdivision within Baias in the regional dataset. Moreover, finer‐scale patterns revealed additional heterogeneity within catchments. Both analyses recovered clear spatial subdivision in Arantzazu and Baias, whereas Añarbe exhibited a more complex pattern. In contrast, the DAPC groups inferred in Agauntza were not spatially associated with sampling sites, and Izki was best represented by a single group. Because these analyses were conducted independently within each catchment, the resulting patterns were not driven by the strong differentiation among catchments detected in the regional analysis. Such within‐catchment structure may arise from multiple causes, including environmental heterogeneity among tributaries or recent fragmentation that restricts movement (Vähä et al. 2007). However, pairwise differences in water physicochemistry showed no detectable additional association with genetic differentiation in our MLPE analysis, suggesting that this component of environmental heterogeneity was unlikely to be a major driver of the observed within‐catchment genetic patterns. The agreement between STRUCTURE and DAPC therefore strengthens the evidence for spatially coherent subdivision in Arantzazu and Baias, while the less consistent patterns in Añarbe and Agauntza should be interpreted more cautiously.

We do not have a definitive explanation for the differences in genetic structure among catchments. In Arantzazu, the reservoir located between Site 3 and the upstream sites may have contributed to the relative isolation of Site 3, although a direct causal relationship cannot be established from our data. Indeed, the dam that forms the reservoir was built on a natural waterfall that would anyway be an important barrier for fish. The pattern observed in Baias is less straightforward. The first artificial barrier encountered when moving upstream from the most downstream sampling site was located between Sites 6 and 7, coinciding spatially with the division between the two genetic clusters. However, additional dams were also present further upstream, but these did not correspond to similarly clear genetic discontinuities. This contrast may reflect differences in barrier age and functional permeability. Some structures may be too recent for detectable differentiation to have developed, whereas others may remain partially passable because of their slope, structural configuration, sluice gates, bypass channels or other potential passage routes. Permeability may also vary through time because high‐discharge events can create temporary opportunities for fish passage (Jamonneau et al. 2026). Therefore, the observed genetic structure cannot be attributed solely to individual dams. Differences among catchments may instead reflect the combined influence of barrier age, structural characteristics and functional permeability, local hydrological regimes, the spatial configuration of tributaries and catchment‐specific demographic histories. In addition, because natural and artificial barriers were considered together in the landscape‐genetic analyses, their relative contributions to the observed genetic differentiation could not be disentangled. More detailed information on barrier construction dates, physical characteristics, alternative passage routes and hydrological conditions would be required to identify why some barriers correspond to genetic discontinuities whereas others do not.

To evaluate which isolation‐related factors contributed to these within‐catchment genetic patterns, we analysed how genetic differentiation varied with river distance, the number of obstacles between sites, and their cumulative height.

Our results showed that the number of obstacles better predicted genetic differentiation than river distance, cumulative obstacle height or water‐quality differences. Consistent with the broader relevance of fragmentation in these river networks, an independent metabarcoding study conducted at the same sampling sites also identified taxon‐specific biological responses associated with river fragmentation (Garrastatxu et al., unpublished data). For *P. bigerri*, the stronger association with barrier number suggests that the frequency of barriers can produce widespread isolation by subdividing habitat into many small, semi‐isolated segments, even when individual barriers are relatively small (compared to large dams and reservoirs) (Consuegra et al. 2021). Consequently, they can act as selective filters (Pavlov et al. 2020; Podgorniak et al. 2016): although they do not completely block gene flow, these structures tend to impede the movement of smaller or less mobile individuals, potentially producing size‐ or mobility‐biased passage and, consequently, altering population composition and evolutionary trajectories (Jones et al. 2021; Parasiewicz et al. 2023).

The genetic consequences of these filters are exacerbated when effective population sizes fall below critical thresholds (Ne < 100, a threshold approached by several estimates in our dataset), as genetic erosion and demographic stochasticity accelerate (Coleman et al. 2018). Such erosion has been shown to reduce temporal stability in fish biomass, leading to cascading effects on population resilience and ecosystem functioning, sometimes with stronger consequences than the loss of species (Fargeot et al. 2025; Prunier, Chevalier, et al. 2023). Beyond shaping spatial genetic structure, fragmentation also influences demographic stability, increasing the likelihood and severity of bottlenecks when disturbances occur.

The heterozygosity‐excess test implemented in BOTTLENECK detected evidence of a recent reduction only in Izki and provided no significant evidence of heterozygosity excess in the remaining populations. This apparent discrepancy with the DIYABC‐RF results is not necessarily contradictory because the two approaches target different demographic signatures. BOTTLENECK is designed to detect the transient excess of heterozygosity that may follow a recent reduction in effective population size, but this signal declines as mutation–drift equilibrium is re‐established and may be difficult to detect when the reduction occurred many generations ago or was gradual or moderate (Cornuet and Luikart 1996; Zachariah Peery et al. 2012). Therefore, the absence of significant heterozygosity excess should not be interpreted as evidence that no demographic reduction occurred. Given that the reductions supported by DIYABC‐RF were inferred to have occurred approximately 100–200 generations ago, the transient signal targeted by BOTTLENECK may have weakened or disappeared, although this explanation cannot be demonstrated directly from our data. BOTTLENECK therefore indicates that most populations do not retain a detectable signature of a very recent bottleneck, whereas DIYABC‐RF evaluates broader alternative demographic histories and supports a post‐medieval reduction in some populations.

In our system, DIYABC‐RF analyses did not support a single demographic trajectory across all populations but, instead, revealed population‐specific demographic histories. A post‐medieval demographic reduction, likely anthropogenic in origin, was signalled for several populations, particularly Izki and Arantzazu, and to a lesser extent Cluster 2 in Baias and Agauntza. However, this pattern was not universal: Añarbe was best explained by a scenario involving a historically large population with no bottleneck, whereas Cluster 1 in Baias was assigned to a scenario of persistently small effective population size, although with low posterior support. Therefore, rather than indicating a widespread demographic collapse across the whole study system, the DIYABC‐RF results suggest that recent anthropogenic bottlenecks affected some populations, while others may have retained demographic stability or remained small in the long term. In the populations where a post‐medieval bottleneck was supported, several human pressures could have contributed to the inferred decline, including habitat fragmentation by dams and weirs, pollution, hydrological alteration and overexploitation (Arthington et al. 2016; Coleman et al. 2018; Valenzuela‐Aguayo et al. 2020). This interpretation is consistent with historical evidence from northern Iberia, where the late medieval and early modern periods were characterised by population growth, intensive land‐use change, expanding fisheries, and increased construction of dams and weirs for mills and smelting activities. Historical records also document strong exploitation of freshwater fish during this period (Almeida et al. 2023).

Although bottlenecks can result from a variety of demographic or environmental factors, their genetic consequences become more severe when they take place in fragmented landscapes. Such bottlenecks can cause rapid allele loss and reduce adaptive potential because, when Ne is small, genetic drift becomes stronger relative to selection, and allele persistence becomes increasingly stochastic, potentially compromising long‐term population persistence (Bijlsma and Loeschcke 2012; Kardos et al. 2021). Although these estimates should be interpreted cautiously given the limited and uneven sample sizes, the low Ne, He and Ar values observed in some populations are compatible with demographic reductions or persistently small population sizes, but are not by themselves diagnostic of a bottleneck event. Comparable responses have been documented in other freshwater fishes. Instream barriers have produced strong differentiation and markedly reduced Ne in *Oncorhynchus clarkii clarkii* (Wofford et al. 2005), while similar effects have been reported for *Salmo trutta* in northern Iberian catchments (González‐Ferreras et al. 2022).

### 4.1 Study Limitations and Future Directions

Several limitations should be considered when interpreting our results. First, sample sizes varied markedly among catchments (20–124 individuals), which may have reduced the precision of allele‐frequency estimates and the probability of detecting low‐frequency alleles in the smallest samples (Hale et al. 2012). Although allelic richness was standardised by rarefaction and no conclusions were based on counts of rare or private alleles, rarefaction does not eliminate uncertainty in allele‐frequency estimates. Unequal sampling may also influence clustering patterns inferred by STRUCTURE (Meirmans 2018). We therefore interpreted the regional clustering cautiously and only alongside within‐catchment analyses and, where applicable, spatial evidence supporting the inferred subdivisions. The broad agreement between STRUCTURE and de novo DAPC for the principal regional patterns and the spatial subdivision of Arantzazu and Baias reduces, but does not eliminate, this uncertainty. Conversely, DAPC patterns based on sites with very small sample sizes should be considered primarily descriptive.

Second, although the 26 microsatellite loci retained in this study were sufficiently polymorphic to identify broad regional differentiation and marked genetic subdivision within some catchments, they provided more limited genome‐wide coverage than current approaches based on thousands of SNPs. Nevertheless, the number of loci is not directly comparable between marker types because microsatellites are multiallelic and highly polymorphic, whereas individual SNPs are generally biallelic. Comparative analyses in freshwater fishes have shown that microsatellites and genome‐wide SNPs can yield highly congruent spatial patterns of genetic diversity, isolation by distance and population structure, although SNP datasets generally provide more precise parameter estimates (Prunier, Veyssière, et al. 2023). Thus, the main limitation of our marker panel concerns the precision and resolution of quantitative estimates rather than its ability to detect pronounced genetic subdivision. Estimates of weak differentiation, contemporary effective population size, demographic timing and posterior demographic parameters may have limited precision and may be sensitive to stochastic variation among loci and to the mutation models assumed for microsatellites. This limitation is particularly relevant where posterior distributions were broad or alternative demographic scenarios received similar support. We therefore consider the broad patterns of regional structure and strong within‐catchment subdivision to be more robust than the exact estimates of population size or the timing and magnitude of demographic changes. Genome‐wide SNP data could provide more precise estimates and potentially greater power to resolve subtle population structure, estimate contemporary migration and discriminate between recent and older demographic processes.

Third, although differences in water physicochemistry were explicitly incorporated into the MLPE analysis and did not explain additional variation in genetic differentiation, other potentially relevant environmental, hydrological, habitat and barrier‐specific characteristics were not considered. Variables such as discharge, habitat availability, productivity, local disturbance and temporal environmental variability may affect population abundance, effective population size, dispersal and the suitability of river reaches for minnows. Their contribution to the observed genetic patterns therefore cannot be excluded. Moreover, although our barrier inventory described barrier origin, type and height, it did not provide a complete characterisation of the functional passability of each structure. Furthermore, because natural and artificial barriers were considered jointly in the landscape‐genetic analyses, we cannot disentangle their relative contributions to the observed genetic differentiation. Characteristics such as barrier age, slope, structural configuration, downstream drop and the presence of side channels, fishways, sluice gates or other potential passage routes may determine the extent to which an individual barrier restricts movement. Barrier age is also relevant because the absence of a genetic discontinuity may indicate either that a structure remains partially permeable or that insufficient time has elapsed for differentiation to become detectable.

Accordingly, the positive association between the number of barriers and genetic differentiation should not be interpreted as demonstrating that barriers were the sole cause of the observed spatial patterns. Although measured differences in water physicochemistry did not account for this relationship, unmeasured environmental, habitat or hydrological variation correlated with network position or barrier distribution may still have contributed. Future landscape‐genetic studies should integrate genome‐wide markers with temporally replicated environmental, hydrological, habitat and barrier‐passability data and detailed descriptions of individual barriers, including their age, slope, structural configuration, available passage routes and position within the river network. Such information would help distinguish the cumulative effect of barrier abundance from the effects of barrier‐specific permeability and identify the structures most likely to constrain gene flow.

### 4.2 Management Implications

From a management perspective, our findings highlight the importance of considering the cumulative effect of obstacles across river networks, as numerous barriers can substantially reduce connectivity even when no single barrier fully explains the observed genetic structure. However, restoration priorities should not be based on barrier number or height alone. Genetic discontinuities and network position should be considered together with barrier‐specific characteristics, hydrological conditions and the availability of alternative passage routes to identify the structures that most strongly restrict movement. The removal or mitigation of strategically selected barriers can therefore yield disproportionate benefits by reconnecting isolated habitat segments and facilitating gene flow (Branco et al. 2014). Where complete removal is not feasible, modifying structures to improve passage or maintaining hydrological conditions that provide effective passage opportunities may also enhance functional connectivity. This is particularly critical in headwater streams, which are both highly vulnerable to fragmentation and central to maintaining biodiversity at the network scale (Finn et al. 2011). Prioritising actions that enhance longitudinal connectivity is thus essential to buffer populations against further genetic erosion and demographic instability.

In parallel, and in line with current conservation frameworks, carefully planned translocations could also play a complementary role in enhancing genetic diversity and adaptive potential. Following the guidelines of Weeks et al. (2011), translocations among subpopulations—and, where appropriate, even among basins—could support genetic rescue, increase adaptive capacity and provide insurance against future environmental change, particularly where physical reconnection is not feasible or would not provide sufficiently rapid demographic reinforcement. However, movements among historically isolated catchments would require especially cautious evaluation of genetic compatibility, environmental matching, local adaptation and the risk of disrupting differentiated gene pools. Genetic data, in combination with barrier inventories and network analyses, can help identify which obstacles would yield the greatest improvements in connectivity if removed and which subpopulations are the most appropriate donors and recipients, ensuring that such actions maximise genetic rescue while minimising associated risks. Together, these strategies offer a framework for mitigating the long‐term genetic consequences of fragmentation and for promoting the resilience of *P. bigerri* populations under ongoing environmental change.

## 5 Conclusions

Our study shows that the genetic structure of the Pyrenean minnow reflects processes operating at nested spatial and temporal scales. Long‐term isolation among catchments remains the dominant source of regional differentiation, whereas complementary STRUCTURE and DAPC analyses revealed additional within‐catchment subdivision, particularly in Arantzazu and Baias. At this finer scale, genetic differentiation was more strongly associated with the number of intervening barriers than with their cumulative height, river distance or water‐quality differences, indicating that repeated barriers distributed across the network can collectively restrict functional connectivity. Demographic analyses further revealed population‐specific histories, with post‐medieval reductions receiving support in several, but not all, populations. Together, these findings show that contemporary fragmentation can reinforce older patterns of isolation without producing uniform genetic responses across river networks. Combining genetic evidence with detailed information on barrier passability, hydrology and network position will allow restoration strategies to move beyond simply counting obstacles and towards identifying those whose mitigation would produce the greatest gains in connectivity. Such targeted actions offer a practical route to preserving genetic diversity, evolutionary potential and the long‐term resilience of *P. bigerri* populations in fragmented rivers.

## Author Contributions

**Jon Garrastatxu:** conceptualization (equal), data curation (equal), investigation (equal). **Maite Arroita:** conceptualization (equal), data curation (equal). **Simon Blanchet:** conceptualization (equal), data curation (equal), formal analysis (equal). **Arturo Elosegi:** conceptualization (equal), data curation (equal). **Jérôme G. Prunier:** conceptualization (equal), data curation (equal), formal analysis (equal). **Aitor Larrañaga:** conceptualization (equal), data curation (equal), formal analysis (equal).

## Funding

This project was funded by the Spanish Ministry of Science, Innovation and Universities (PID2020‐115830GB‐100). This research was also supported by the Basque Government (Consolidated Research Group: Stream Ecology 7‐CA‐18/10, IT1471‐22) and the University of the Basque Country (predoctoral fellowship PIF21/22, J.G.). S.B. and J.G.P. were financially supported by a grant from the French Office for Biodiversity.

## Conflicts of Interest

The authors declare no conflicts of interest.

## Supporting information


**Supplementary File A:** Excel document with the summaries of the Evanno methods, and the substructure for each population.

**Supplementary File B:** Excel document with the confusion matrices with the four scenarios tested for each population.

**Supplementary File C:** Excel document summarising the missing‐data filtering procedure for the SSRseq dataset, including locus‐ and individual‐level filtering, the applied missing‐data thresholds, and the loci and individuals retained or excluded to obtain the final dataset of 280 individuals genotyped at 26 loci.

**Table S1:** Number of samples analysed at each sampling site within the Baias, Izki, Arantzazu, Añarbe and Agauntza catchments. Site numbers correspond to those shown in the sampling‐location maps.
**Table S2:** Number and cumulative height (m) of natural and artificial barriers recorded in the Baias, Izki, Arantzazu, Añarbe and Agauntza catchments. Percentages indicate the proportion of artificial barriers relative to the total number of barriers and their contribution to the total cumulative barrier height within each catchment.
**Figure S1:** BIC profiles for de novo *K*‐means clustering conducted for (A) the complete regional dataset, (B) the Atlantic drainage, (C) the Mediterranean drainage, and independently within (D) Arantzazu, (E) Agauntza, (F) Añarbe, (G) Baias and (H) Izki. Solid vertical lines indicate the selected number of clusters (*K*). The selected solutions were *K* = 6 for the regional dataset, *K* = 3 for the Atlantic drainage, *K* = 3 for the Mediterranean drainage, *K* = 2 for Arantzazu, *K* = 2 for Agauntza, *K* = 3 for Añarbe, *K* = 2 for Baias and *K* = 1 for Izki.
**Figure S2:** De novo discriminant analyses of principal components (DAPC) conducted independently within (A) Arantzazu, (B) Agauntza, (C) Añarbe and (D) Baias. Individuals are coloured according to sampling site, which was used only after de novo clustering to describe the spatial composition of the inferred genetic groups. Izki is not shown because *K* = 1 was retained and DAPC is not applicable in the absence of between‐group variation.
**Figure S3:** Prior‐scenario checking for the DIYABC‐RF demographic analyses. Principal component analyses showing the distribution of datasets simulated under the four demographic scenarios relative to the observed genetic dataset for (A) Baias 1–6, (B) Baias 7–10, (C) Izki, (D) Agauntza, (E) Añarbe and (F) Arantzazu. Each point represents a simulated dataset, colours indicate demographic scenarios, and the observed dataset is shown in red. In all analyses, the observed dataset fell within the multivariate space covered by the simulations. The first two principal components explained approximately 94% of the total variation.

## Data Availability

The data underlying this article are available in the Dryad Digital Repository at https://doi.org/10.5061/dryad.fbg79cp9h.
