# Supplementary material for: Effects of Historical Isolation and Current River Fragmentation on the Genetic Structure of a Freshwater Fish
Source: Ecol Evol. 2026 Nov 9;16(11):e74633. doi: 10.1002/ece3.74633 (PMC13748948; doi:10.1002/ece3.74633)
Supplement: Supplementary file 1 [file ECE3-16-e74633-s001.docx]

**Supporting Information**

**File S1:** Excel document with the summaries of the evanno methods, and the substructure for each population.

**File S2:** Excel document with the confusion matrices with the 4 scenarios tested for each population:

**File S3:** Excel document summarizing the missing-data filtering procedure for the SSRseq dataset, including locus- and individual-level filtering, the applied missing-data thresholds, and the loci and individuals retained or excluded to obtain the final dataset of 280 individuals genotyped at 26 loci.

**Table S1:** Number of samples analysed at each sampling site within the Baias, Izki, Arantzazu, Añarbe, and Agauntza catchments. Site numbers correspond to those shown in the sampling-location maps.

| Catchment | Site | Nº of samples |
| --- | --- | --- |
| Baias | 1 | 14 |
|  | 2 | 9 |
|  | 3 | 4 |
|  | 4 | 14 |
|  | 5 | 13 |
|  | 6 | 14 |
|  | 7 | 15 |
|  | 8 | 14 |
|  | 9 | 13 |
|  | 10 | 14 |
| Izki | 7 | 2 |
|  | 8 | 15 |
|  | 10 | 15 |
| Arantzazu | 3 | 3 |
|  | 8 | 3 |
|  | 9 | 5 |
|  | 10 | 9 |
| Añarbe | 1 | 12 |
|  | 4 | 15 |
|  | 5 | 5 |
|  | 6 | 6 |
|  | 10 | 5 |
| Agauntza | 1 | 15 |
|  | 4 | 6 |
|  | 5 | 1 |
|  | 7 | 9 |
|  | 9 | 15 |
|  | 10 | 15 |

**Table S2:** Number and cumulative height (m) of natural and artificial barriers recorded in the Baias, Izki, Arantzazu, Añarbe, and Agauntza catchments. Percentages indicate the proportion of artificial barriers relative to the total number of barriers and their contribution to the total cumulative barrier height within each catchment.

| Catchment | Type | Total barriers | Total Height |
| --- | --- | --- | --- |
| Baias | Natural  Artificial  % Artificial | 17  8  32% | 38.5  22.5  37% |
| Izki | No barriers | 0 | 0 |
| Arantzazu | Natural  Artificial | 7  3 | 19.5  30 |
|  | % Artificial | 30% | 61% |
| Añarbe | Natural  Artificial  % Artificial | 1  2  67% | 3  12  80% |
| Agauntza | Natural | 12 | 70 |
|  | Artificial | 2 | 21 |
|  | % Artificial | 14% | 23% |

**Figure S1:** BIC profiles for de novo K-means clustering conducted for (A) the complete regional dataset, (B) the Atlantic drainage, (C) the Mediterranean drainage, and independently within (D) Arantzazu, (E) Agauntza, (F) Añarbe, (G) Baias, and (H) Izki. Solid vertical lines indicate the selected number of clusters (K). The selected solutions were K = 6 for the regional dataset, K = 3 for the Atlantic drainage, K = 3 for the Mediterranean drainage, K = 2 for Arantzazu, K = 2 for Agauntza, K = 3 for Añarbe, K = 2 for Baias, and K = 1 for Izki.

**Figure S2:** De novo discriminant analyses of principal components (DAPC) conducted independently within (A) Arantzazu, (B) Agauntza, (C) Añarbe, and (D) Baias. Individuals are coloured according to sampling site, which was used only after de novo clustering to describe the spatial composition of the inferred genetic groups. Izki is not shown because K = 1 was retained and DAPC is not applicable in the absence of between-group variation**.**

**Scenario 1** – large constant population;
**Scenario 2** – large population with recent bottleneck;
**Scenario 3** – large population with ancient bottleneck;
**Scenario 4** – small constant population.

**Figure S3:** Prior-scenario checking for the DIYABC-RF demographic analyses. Principal component analyses showing the distribution of datasets simulated under the four demographic scenarios relative to the observed genetic dataset for (A) Baias 1–6, (B) Baias 7–10, (C) Izki, (D) Agauntza, (E) Añarbe, and (F) Arantzazu. Each point represents a simulated dataset, colours indicate demographic scenarios, and the observed dataset is shown in red. In all analyses, the observed dataset fell within the multivariate space covered by the simulations. The first two principal components explained approximately 94% of the total variation.
